# The complete plastome sequences of invasive weed *Parthenium hysterophorus*: genome organization, evolutionary significance, structural features, and comparative analysis

**DOI:** 10.1038/s41598-024-54503-0

**Published:** 2024-02-18

**Authors:** Lubna, Sajjad Asaf, Rahmatullah Jan, Saleem Asif, Saqib Bilal, Abdul Latif Khan, Ahmed N. Al-Rawahi, Kyung-Min Kim, Ahmed AL-Harrasi

**Affiliations:** 1 Natural and Medical Sciences Research Center, University of Nizwa, 616 Nizwa, Oman; 2 Department of Applied Biosciences, Kyungpook National University, Daegu, 41566 Republic of Korea; 3 Department of Engineering Technology, University of Houston, Sugar Land, TX 77479 USA

**Keywords:** Weed, Chloroplast, Divergence, Phylogenetic, Synteny, Computational biology and bioinformatics, Molecular biology

## Abstract

*Parthenium hysterophorus*, a globally widespread weed, poses a significant threat to agricultural ecosystems due to its invasive nature. We investigated the chloroplast genome of *P. hysterophorus* in this study. Our analysis revealed that the chloroplast genome of *P. hysterophorus* spans a length of 151,881 base pairs (bp). It exhibits typical quadripartite structure commonly found in chloroplast genomes, including inverted repeat regions (IR) of 25,085 bp, a small single copy (SSC) region of 18,052 bp, and a large single copy (LSC) region of 83,588 bp. A total of 129 unique genes were identified in *P. hysterophorus* chloroplast genomes, including 85 protein-coding genes, 36 tRNAs, and eight rRNAs genes. Comparative analysis of the *P. hysterophorus* plastome with those of related species from the tribe Heliantheae revealed both conserved structures and intriguing variations. While many structural elements were shared among the species, we identified a rearrangement in the large single-copy region of *P. hysterophorus*. Moreover, our study highlighted notable gene divergence in several specific genes, namely *mat*K, *ndh*F, *clp*P, *rps*16, *ndh*A, *rps*3, and *ndh*D. Phylogenetic analysis based on the 72 shared genes placed *P. hysterophorus* in a distinct clade alongside another species, *P. argentatum*. Additionally, the estimated divergence time between the *Parthenium* genus and *Helianthus* (sunflowers) was approximately 15.1 million years ago (Mya). These findings provide valuable insights into the evolutionary history and genetic relationships of *P. hysterophorus*, shedding light on its divergence and adaptation over time.

## Introduction

The sunflower family, also known as Asteraceae or Compositae, is renowned for its remarkable diversity in the plant kingdom. It encompasses approximately 25,000 to 35,000 species, which are distributed across the globe and makeup around 10% of all flowering plants^1,2^. The family Asteraceae comprises numerous significant crops such as lettuce, sunflower, and artichoke, as well as a variety of ornamental plants like marigolds and dahlias^1,2^. However, it also includes several weed species like dandelions, *Parthenium,* and certain thistles^1^.

*P. hysterophoruss,* belonging to the Asteraceae family, is a highly invasive weed present in more than 50 countries. It has gained significant notoriety globally as one of the most troublesome weed species. Its detrimental characteristics include its remarkable seed production of approximately 20,000 seeds per plant, fast germination, fast growth rate, and capacity to release chemicals (allelopathy) that inhibit the growth of other plants^3^. The seeds of *P. hysterophorus* can germinate across a wide range of temperatures, but their germination is primarily influenced by the moisture content of the soil^4–6^. Exposure to *P. hysterophorus* can lead to severe dermatitis, hay fever, and other allergic reactions in animals and humans^7^. This weed thrives in areas with high light intensity^4^ and increased nitrogen levels^8^. *P. hysterophorus* has been known to cause significant crop yield reductions, ranging from 40 to 97%, and can also act as a secondary host for various crop diseases^3^. To effectively manage *P. hysterophorus*, both chemical^9^ and biological control methods have been found to be successful^4,10^. Additionally, cultivating highly competitive crops has proven to be highly effective in suppressing the emergence and initial growth of *P. hysterophorus*^11,12^. The genetic makeup of an invasive species can undergo alterations as it spreads from its original habitat to new locations, resulting in shifts in the distribution of genetic diversity among and within different populations.^13,14^. In 2020, a study examining the genetic diversity and population structure of *P. hysterophorus* in various regions of Jammu and Kashmir. The findings revealed that there was a limited level of overall genetic diversity, as determined through the utilization of ISSR markers^15^. Foran invasive species to thrive in a new environment, having a high degree of phenotypic plasticity, which helps it adapt to various selection pressures, can be more crucial than relying solely on the slow accumulation of genetic variability over time^16^.

Chloroplasts (cp), specializd organelles found in plants and algae, are vital for the energy production of these organisms through photosynthesis. They evolved from cyanobacteria due to endosymbiosis^17,18^. These organelles have their genetic replication mechanism and can transcribe their own genome. Additionally, they exhibit maternal inheritance, meaning they are passed down from the mother to the offspring^17,18^. The plastomes of flowering plants, also known as angiosperms, are usually around 120 to 160 kb in size. They have a unique structure consisting of four parts: two single-copy regions called the long single copy (LSC) and the short single copy (SSC), which are separated by two inverted repeats (IRA and IRB)^19^. In the Asteraceae family, all examined plastomes are around 150 kb long and exhibit the anticipated quadripartite organization. These genomes consist of approximately 80 protein-coding genes, along with four ribosomal RNAs (rRNAs) and 30 transfer RNAs (tRNAs)^20^. Although large-scale changes in plastid DNA structure are infrequent among land plants, certain plant families, such as Geraniaceae, Fabaceae, and Ericaceae, demonstrate a range of intriguing plastome rearrangements. These rearrangements encompass expansions, contractions, inversions, or even the loss of an inverted repeat (IR)^21^. Most Asteraceae plastomes, excluding those belonging to the Barnadesioideae subfamily, comprising roughly 100 species, showcase a distinct and notable structural trait. This feature involves a double inversion within the plastid DNA, setting them apart from their Barnadesioideae counterparts^22^. These inversions, located in the LSC region, consist of a larger inversion, approximately 22.8 kb, which contains a second inversion, approximately 3.3 kb in length. These inversions have been confirmed through different sequencing methods, including Sanger and next-generation sequencing (NGS)^22,23^. Further research incorporating a wider range of species is required to gain additional insights into the structural variations of Asteraceae plastomes^22^.

Over the past two decades, the plastid genome sequence has served as a valuable resource for DNA barcoding in plant identification^24^, and it can also contribute to the development of informative markers for population studies^25^. The significance of the plastid genome extends to phylogenetic analysis, DNA barcoding, photosynthesis research, and, more recently, transcriptomics^26^, resulting in the sequencing of an ever-growing number of complete plastomes. With the advent of next-generation sequencing technologies and their decreasing costs, large-scale genomic data generation for multiple species, including plastid DNA, has become feasible. User-friendly de novo assembly bioinformatics tools such as NOVOPlasty^27^ and SOAPdenovo2^28^ have simplified plastome reconstruction. Consequently, the plastid genomes of several Asteraceae species have been sequenced and made publicly accessible. However, the existing genomic data suffers from a fragmented and uneven taxonomic representation, necessitating the acquisition of additional data to analyze plastome diversity within the family comprehensively. Since the publication of the first complete chloroplast genome of *Nicotiana tabacum* (source:^29^, more than 3,700 complete plastid genomes have been sequenced and studied^30^. Plastid genomes have been sequenced in the Asteraceae family, including *Guizotia abyssinica*^31^, *Helianthus annuus*^32^, and *Parthenium argentatum*^23^. A comprehensive examination was conducted previously on the plastomes of 36 species belonging to various subfamilies and tribes within the Asteraceae family^2^.

In this study, we have successfully sequenced and analyzed the entire plastome sequence of *P. hysterophorus* using advanced Illumina high-throughput sequencing technology. Furthermore, we compared these sequences with twelve previously sequenced plastomes from the Helaianthae tribe. This comprehensive dataset of plastomes will serve as valuable genetic resources for conducting population and phylogenetic studies on *P. hysterophorus*.

## Results

### General features of the *P. hysterophorus* plastome

**Figure 1:**
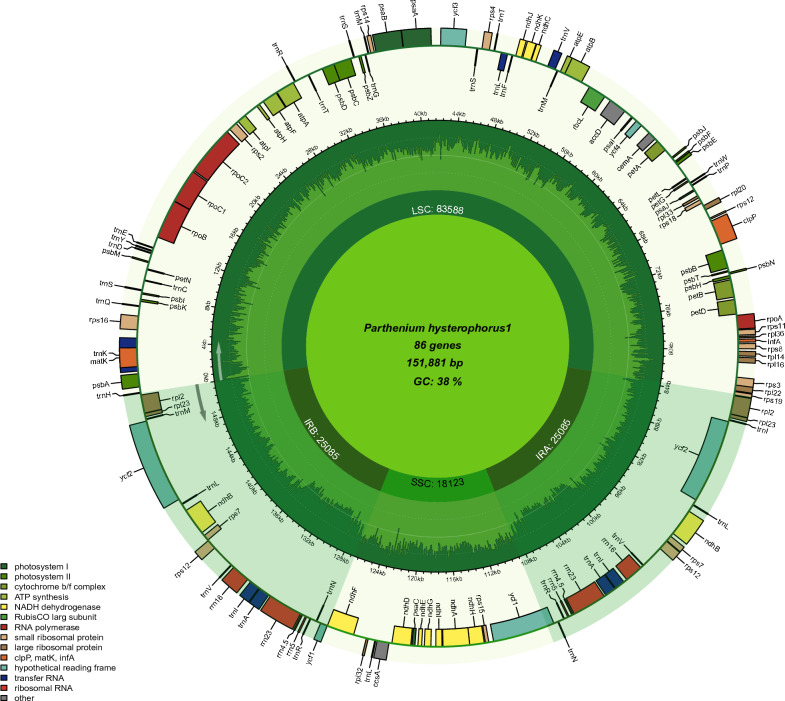
Genome map of the *P. hysterophorus* plastomes. The extent of the IR regions is represented by dark colors, which divide the cp genome into large (LSC) and small (SSC) single copy regions. Genes drawn inside the circle are transcribed clockwise, whereas those outside of the circle are transcribed counter-clockwise. Genes belonging to different functional groups are color coded. The light green in the inner circle corresponds to the GC content, whereas the dark green corresponds to the AT content. The circular chloroplast genome map was drawn using the online program Chloroplot ((https://irscope.shinyapps.io/Chloroplot/).

The circular map represents the entire structure of the *P. hysterophorus* plastome, which spans 151,881 bp in length. It consists of a duplicated region known as inverted repeats (IR), which accounts for 25,085 bp. These IR regions are positioned on opposite ends of the genome and are separated by two distinct regions: a small single copy (SSC) region measuring 18,052 bp and a large single copy (LSC) region spanning 83,588 bp (Fig. 1 and Table 1). The overall G + C content of the entire chloroplast genome is 37.6%. The GC content of rRNA is greater (55.3%) than other parts of plastome. Among other studied species, *P. argentatum* has the longest genome size of 152,803 bp with 76,636 bp protein-coding regions. *H. annuus* has the shortest (151,104 bp) plastome size among all species (Table 1). In *P. hysterophorus,* there are 129 genes, including 85 genes coding for proteins, eight rRNA genes, and 36 genes for tRNA. The chloroplast (cp) genome contains various protein-coding genes, including 15 genes associated with photosystem II (*psb*A, B, C, D, E, F, H, I, J, K, L, M, T, Z), nine genes encoding large ribosomal proteins (*rpl*2, 14, 16, 20, 22, 23, 32, 33, 36), 11 genes for small ribosomal proteins (*rps*2, 3, 4, 7, 8, 11, 12, 14, 15, 18, 19), five genes related to photosystem I (*psa*A, B, C, I, J), and six genes responsible for ATP synthesis and the electron transport chain (*atp*A, B, E, F, H, I). Notably, the *psb*L gene is not found in the plastome. Similarly, 17 protein-coding genes contained introns, of which three genes (*clp*P, *rps*12, *ycf*3) comprised two introns, while the rest contained only a single intron (Fig S1). The length of the protein-coding region is about 71,064 bp, while those of tRNA and rRNA are 2,713 bp and 9,050 bp, respectively (Table 1).

**Table 1:** Summary of all *P. hysterophorus* and related plastomes.

	P.hy1	P. hy2	P. ar	A. an	A. ar	E. ang	H. an	I. het	T. div	S. int	S. cal	X. sib
Size (bp)	151,881	151,912	152,803	151,330	152,215	151,935	151,104	151,495	151,356	152,058	151,748	151,897
GC contents	37.6	37.6	37.6	37.6	37.6	37.6	37.6	37.6	37.6	37.5	37.5	37.5
SSC (bp)	18,052	18,122	18,843	18,330	17,863	18,159	18,497	21,915	18,400	18,354	18,348	17,901
IR (bp)	25,085	25,093	24,684	24,642	24,929	25,081	24,634	22,875	24,645	25,028	25,065	25,081
PCD ((bp)	71,964	78,741	76,636	77,442	78,531	78,018	77,370	77,475	77,490	77,634	73,203	128,748
tRNA (bp)	2713	2733	1276	2763	2804	2713	2713	2764	2764	3203	2727	2794
rRNA (bp)	9050	9047	4949	9050	9050	9050	9052	9050	9050	9050	9047	9050
Total genes	129	132	129	132	134	131	138	132	132	134	130	133
Protein Coding genes	85	87	85	85	87	85	85	85	85	87	86	87
rRNA	8	8	8	8	8	8	8	8	8	8	8	8
tRNA	36	36	37	37	37	36	43	37	37	37	36	37

P.hy1, *Parthenium hysterophorus* (new); P. hy2, *Parthenium hysterophorus* (old); P. ar, *Parthenium argentatum*; A. an, *Aldama anchusifolia*; A. ar, *Ambrosia artemisiifolia*; E. ang, *Echinacea angustifolia*; H. an, *Helianthus annuus*; I. het, *Iostephane heterophylla*; S. int, *Silphium integrifolium*; S. cal, *Sphagneticola calendulacea*; X. sib, *Xanthium sibiricum.*

### Structural comparison for *P. hysterophorus* plastome with related species

**Figure 2:**
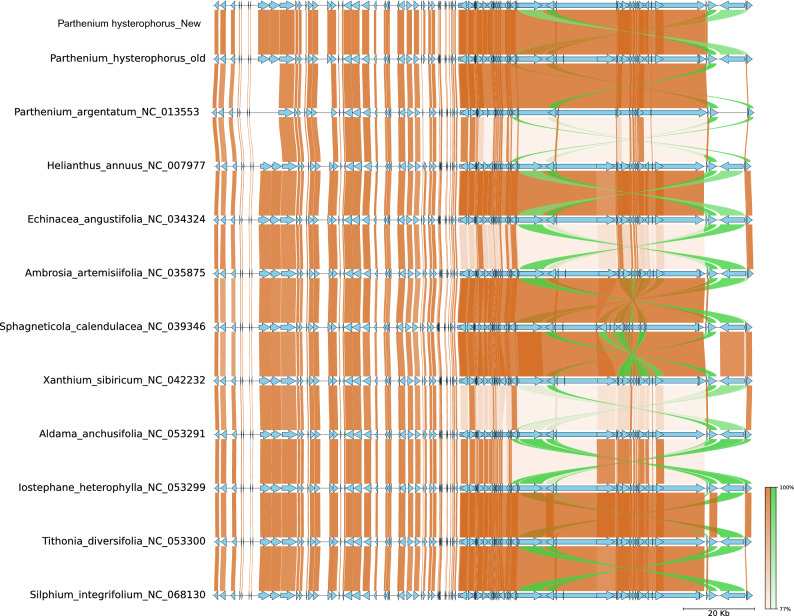
Synteny plot of *P. hysterophorus* plastome with eleven related speceis plastomes. The synteny plot shows normal links with chocolate color, inverted link with lime-green color, and gene feature with sky-blue color.

Structural analysis of *P. hysterophorus* revealed that, like most of other Asteraceae plastomes, it exhibits a high level of sequence similarity and structural conservation (Fig. 2). The analysis has provided evidence supporting the presence of a rearrangement in the LSC region of the genome. This rearrangement involves two inversions: a relatively large inversion spanning approximately 22.8 kilobases (kb) and a smaller inversion nested within the larger one, spanning around 3.3 kb. Notably, this rearrangement is observed across all species belonging to the subfamilies Cichorioideae, Carduoideae, Mutisioideae, and Asteroideae (Fig. 2). Synteny visualizations were utilized to identify similarities and differences among these genomes. The results demonstrated that *P. hysterophorus* is closely related to all the related species from Halianthae and exhibited high levels of synteny and similarity (Fig. 2). However, a comparison with *Arabidopsis,* a model plastome, confirmed the large inversion in the LSC region, as reported above (Fig S2).

**Figure 3:**
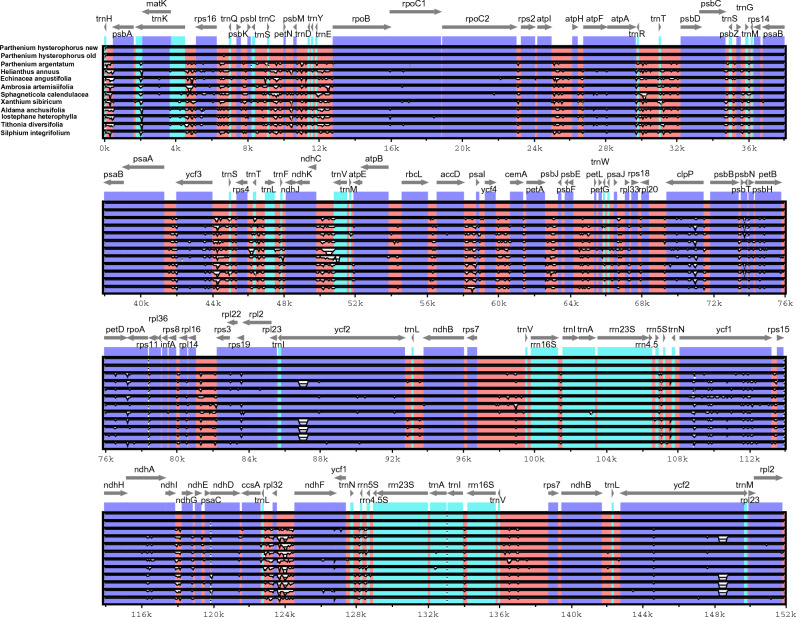
Visual alignment of *P. hysterophorus* and eleven related plastomes (*P. hysterophorus* (old)*, P. argentatum, A. anchusifolia, A. artemisiifolia, E. angustifolia, H. annuus, I. heterophylla, T. diversifolia, S. integrifolium, S. calendulacea and X. sibiricum*) from the Heliantheae tribe. VISTA-based identity plot showing sequence identity among these species, using *P. hysterophorus* as a reference. The vertical scale indicates percent identity, ranging from 50 to 100%. The horizontal axis indicates the coordinates within the chloroplast genome. Arrows indicate the annotated genes and their transcription direction.

**Figure 4:**
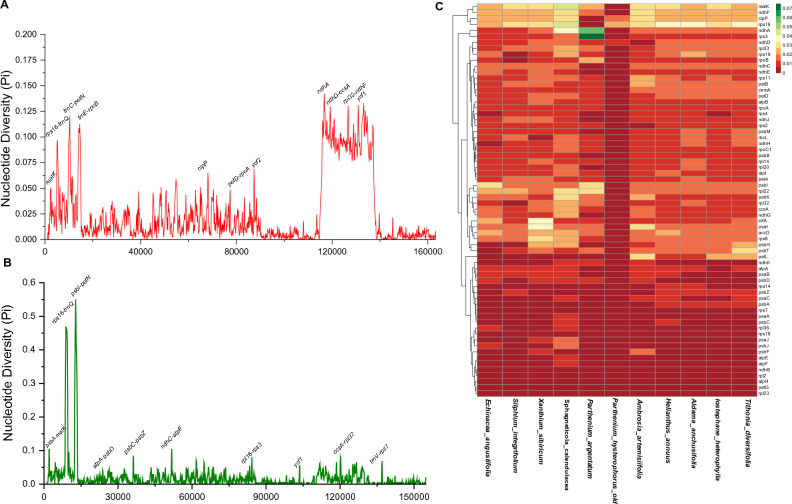
Sliding window analysis of nucleotide variability among the *P. hysterophorus* and related plastomes (window length: 200 bp; step size: 100 bp), **(A)** nucleotide variability between *P. hysterophorus* and *P. argentatum*. **(B)** Nucleotide variability among *P. hysterophorus and related eleven* plastomes from Heliantheae. **(C)** Heatmap showing pairwise sequence distance of 66 genes from of *P. hysterophorus* and related plastomes from Heliantheae.

The availability of multiple complete Asteraceae plastomes offers a valuable opportunity to investigate sequence variations within the family at the genome level. Using the VISTA program and referencing the annotation of *P. hysterophorus*, we aligned and plotted the plastomes of 12 Asteraceae species (Fig. 3). The overall alignment of these genomes reveals a predominantly conservative nature, with limited divergent regions. As observed in other angiosperms, the coding regions exhibit higher conservation levels than the non-coding counterparts. A number of regions are found to show more divergence, including *trnH-psbA*, *matK*, *rps16*-trnE, *trnR-psbD, ndh*C-*trn*V, *ycf*3-*trn*S, *clp*P, *pet*B, *ycf*1, *rpo*A, *rpl*32, and *ndh*F, but the divergence is much more in *A. artemisiifolia*. Similarly, in *P. argentatum* the sequence divergence from *psbl*- *trnC* to *trnE*-*rpoB* is more. The *trnT*, *trnS psaA*-*ndhj* showed more divergence. In *X. sibiricum* and *S. calendulacea* the *trnV* exhibited greater divergence than other species (Fig. 4). On the other hand, *A. artemisiifolia*, *H.annuus*, *A. anchusifolia*, *I. heterophylla* and *T. diversifolia*, from *accD*-*psal* and *trnL*-*ycf2* region showed more divergence (Fig. 4). In *S. calendulacea* the gene from *trnN*-*ndhF* is missing, while in *P. argentatum, trn*N showed significant divergence, *A. artemisiifolia*, *H. annuus*, *A. anchusifolia*, *I. heterophylla,* and *T. diversifolia* also showed high divergence in *ycf2* gene as compared to *P. hysterophorus*. In a pairwise sequence divergence analysis, *P. hysterophorus* exhibited the highest divergence (0.07) with *S. calendulacea* followed by *X. sibiricum* (0.02) and showed the lowest divergence with previously sequenced *P. hysterophorus* (0.00007), followed by *P. argentatum* (0.018) (Table S1).

**Figure 5:**
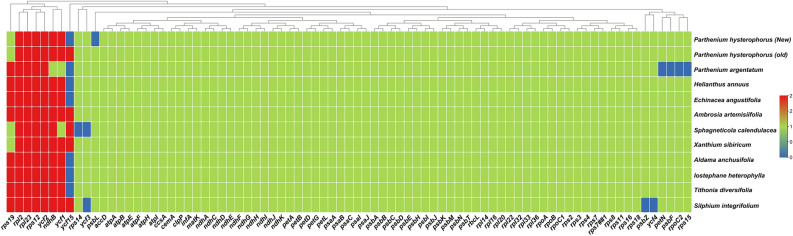
Summary of genes lost across *P. hysterophorus* and related species plastomes. The blue color shows the missing genes, green color shows single genes whereas the red shows the genes duplicated in plastomes.

Furthermore, the values of nucleotide diversity (Pi) were determined in plastomes *P. hysterophorus* and other related species (Fig. 4A). The genomes were aligned in two different groups: (i) One currently sequenced *P. hysterophorus* and *P. argentatum* plastome and (ii) *P. hysterophorus* and all eleven related species plastomes to better evaluate and understand the nucleotide diversity (Pi). The nucleotide diversity (Pi) values within 200 bp window size and 100 bp step size across these plastomes vary from 0 to 0.55 (Fig. 4B) and 0 to 0.14 (Fig. 4A), respectively. Only six variable loci (*trn*C-*pet*N, *trn*E-*rpo*B, *ndh*A, *ndh*D-*ccs*A, *rpl*32-*ndh*F, and *yc*f1) were found with Pi > 0.1 in *P. hysterophorus* with related plastomes while with *P. argentatum* only two loci (*rps*16*-trn*Q and *psb*I*-pet*N) were found with Pi > 0.3 (Fig. 4B). The most divergent genes were *mat*K, *ndh*F, *clp*P, *rps*16, *ndh*A, *rps*3*,* and *ndh*D (Fig. 4C). Surprisingly, the highest divergence was observed in *ndh*A and *rps*3 genes in *P. argentatum*. Likewise, *mat*K, *ndh*F, *clp*P, *rps*16, and *ndh*A genes were found to have higher divergence in *S. calandulaceae* (Fig. 4C). The gene contents of *P. hysterophorus* were compared with related species, and no considerable variation was observed among these plastomes*.* These plastomes contained 85–87 protein-coding genes, eight rRNA genes, and 36–37 tRNA genes. We compared all twelve plastomes and found that the *ycf15* gene was absent in many plastomes, such as *P. hysterophorus, H. annuus, E. angustifolia, A.anchustifolia, I. heterophylla* and *T. diervsifolia* (Fig. 5). Similarly*, ycf*3*, psb*Z*,* and *ycf*4 *genes* were absent in *S. integrifolium* plastome (Fig. 5)*.*

### Contraction and expansion of IRs

**Figure 6:**
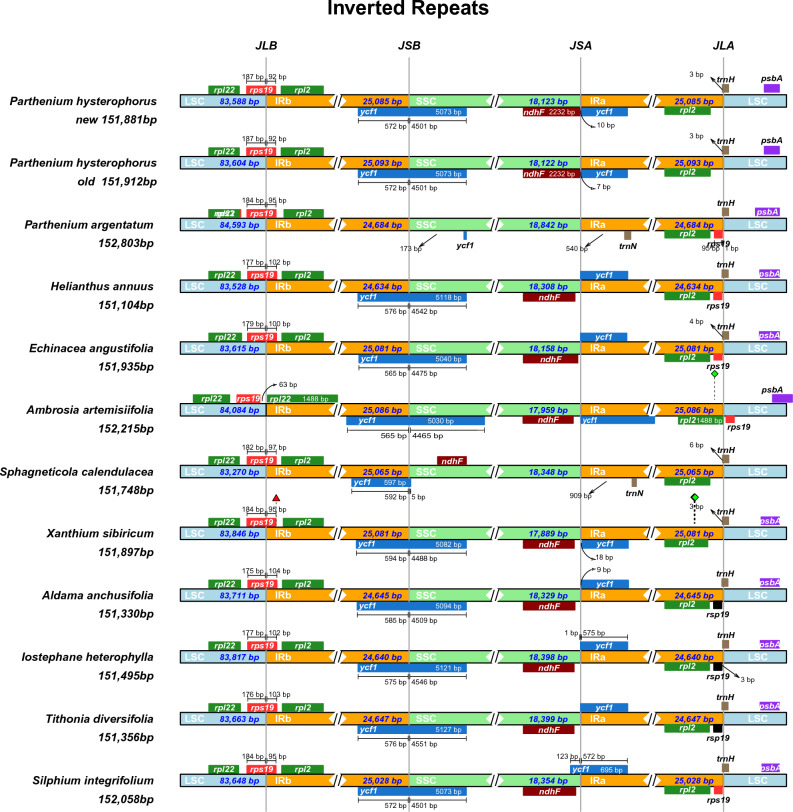
Distances between adjacent genes and junctions of the small single-copy (SSC), large single-copy (LSC), and two inverted repeats (IR) regions among *TP. hysterophorus* and *related* plastomes. Boxes above and below the primary line indicate the adjacent border genes. The Fig is not scaled regarding sequence length and only shows relative changes at or near the IR/SC borders.

The borders of LSC-IRb and SSC-IRa in the plastome of *P. hysterophorus* were compared to 11 other closely related species, including *A. anchusifolia*, *A. artemisiifolia*, *E. angustifolia*, *H. annuus*, *I. heterophylla*, *P. argentatum*, *P. hysterophorus*, *T. diversifolia*, *S. integrifolium*, *S*. *calendulacea,* and *X. sibiricum*. All species had an intact copy of the *rps*19 gene across the LSC/IRb (JLB) border. The *rpl*22 gene is located in the LSC region in all species. The *rps*19 gene passes through JLB junction, and 187 bp occurs on the LSC side in*P. hysterophorus* and 92 bp in *IRb* region, 184 bp on the LSC side in *P. argentatum*, *X. sibiricum,* and *S. integrifolium* and 95 bp in *IRb* region, 177 bp in *H. annuus* and *I. heterophylla in* LSC and 102 bp in *IRb* region in *E. angustifolia* 179 bp in LSC and 100 bp *IRb* region in *S. calendulacea* 182 bp LSC and 97 bp *IRb* region. However, in *A. artemisiifolia rps*19 gene is present in the LSC region 63 bp away from JLB junction (Fig. 6). The *rpl2* gene lies in IRb region just near to *JLB* border. The *ycf1* gene passes through JSB border except in *P. argentatum,* which is located in SSC region, and in *A. artemisiifolia* it passes through JLA border in *S. calendulacea,* the *ycf1* gene is 597 bp in IRb region, and only 5 bp in SSC region. Similarly, the *ndhF* gene is located close to JSA border toward SSC side except in *P. argentatum,* while in *S. calendulacea* it is located near JSB region in SSC region. The *trnH* gene occurs intact with JLA junction toward the LSC region. The *trnN* gene only occurs in *S. calendulacea* in IRa region.

### Repeat sequence analysis

**Figure 7:**
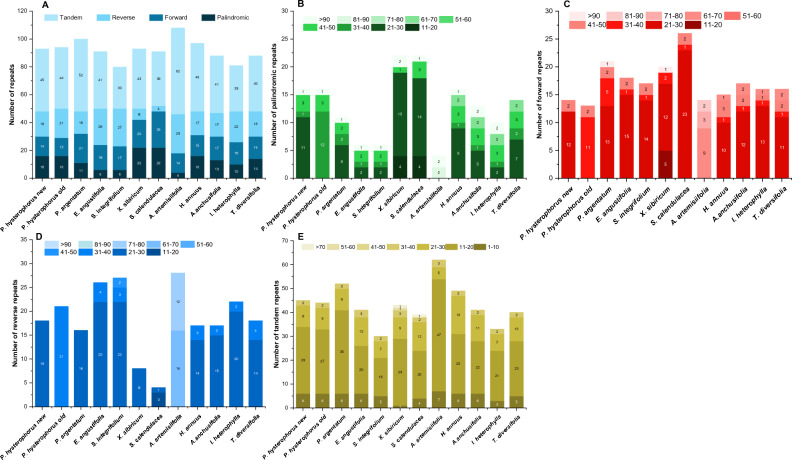
Repetitive sequences in *P. hysterophorus* and eleven related plastomes (**A**) Total number of repetitive sequences. (**B**) Lengthwise frequency of palindromic repeats in plastomes, (**B**) Lengthwise frequency of forward repeats, (**C**) lengthwise frequency of reverse repeats, (**D**) lengthwise frequency of tandem repeats.

Different types of repeats were examined in *P. hysterophorus* and compared with other related species. The result showed that *P. hysterophorus* consists of a total of 16 palindromic repeats, 14 forward repeats and 18 reverse repeats, and 45 tandem repeats. However, in *P. argentatum*, these repeats were 11, 21, 16, and 52, respectively. Among the related plastomes, the highest number of tandem (52) and reverse (28) repeats were found in *A. artemissifolia* (Fig. 7). However, among the other species *X. sibiricum* and *S. calendulacea* possess the highest palindromic and forward repeats, i.e. (22, 22), (20, 26) respectively, while *A. artemisiifolia* comprised the lowest palindromic repeats (4). However, *A. artemisiifolia* comprised the highest reverse repeats (28), followed by *S. integrifolium* 27. On the other hand, *X. sibiricum* and *S. calendulacea* have the lowest reverse repeats, 8 and 4, respectively. In the case of tandem repeats *A. artemisiifolia* comprised the highest number of tandem repeats e.g. 62. However, when we observed the length of different repeats, we found that in the case of palindromic, forward, and reverse repeats, most of the repeats were 21–30 bp long, while in the case of tandem repeats, majority of repeats were 11–20 bp long in all plastomes. In *A. artemisiifolia,* about 16 and 12 repeats were of 61–70 and 71–80 bp in length (Fig. 7).

### Simple sequence repeats (SSRs) analysis

**Figure 8:**
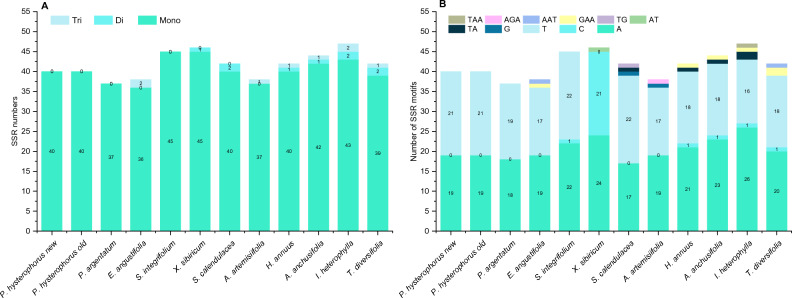
Analysis of the simple sequence repeats (SSRs) in *P. hysterophorus* and eleven related plastomes; (**A**) total number of SSR repeats in genomes; (**B**) frequency of the simple sequence repeat motif in the chloroplast genome of *P. hysterophorus* and and eleven related plastomes.

In *P. hysterophorus* plastome, a total of 40 SSR repeats are detected, and all of them are mononucleotide repeats. The highest number of repeats were observed in *I. heterophylla* (47) with 43 mononucleotides, two dinucleotides, and two trinucleotides and *X. sibiricum* (46) with 45 mononucleotides and one dinucleotide. About 45 SSRs were observed in *S. integrifolium* followed by *A. anchusifolia* (44) and *T. diversifolia* (42). No tetra and pentanucleotide SSRs were detected in any plastome. In *P. hysterophorus*, most mononucleotide SSRs were A (47.5%) and T (52.5%) motifs (Fig. 8). The highest C motif (45%) was observed in *X. sibiricum,* while only one C motif was observed in five species (*S. integrifolium, H. annuus, A. anchusifolia, I. heterophylla,* and *T. diversifolia*) while no C motif was detected in *Parthenium* species plastomes. Only one dinucleotide with AT motif was observed in *X. sibiricum,* while one TA motif was observed in *S. calendulacea, H. annuus,* and *A. anchusifolia* while two TA motifs were observed in *I. heterophylla* plastome. Similarly, two trinucleotide motifs (GAA) were observed in *T. diversifolia*.

### Phylogenetic analysis

**Figure 9:**
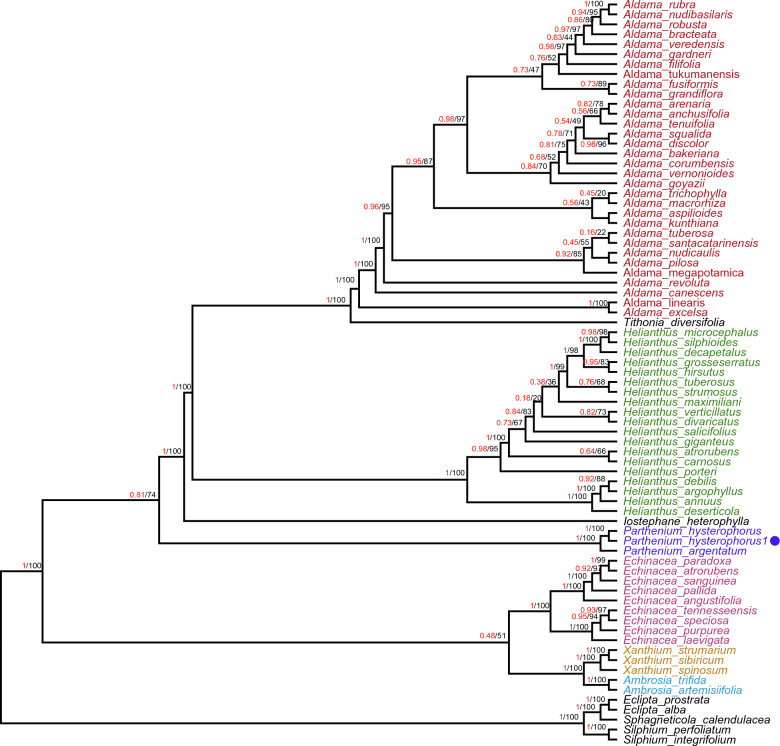
Phylogenetic trees were constructed from 72 commonly shared genes among 75 members of the Heliantheae tribe, representing 11 different genera using different methods, Bayesian inference (BI) and maximum likelihood (ML). Numbers above the branches are the posterior probabilities of BI and bootstrap values of ML. Dot represent the position for *P. hysterophorus*.

**Figure 10:**
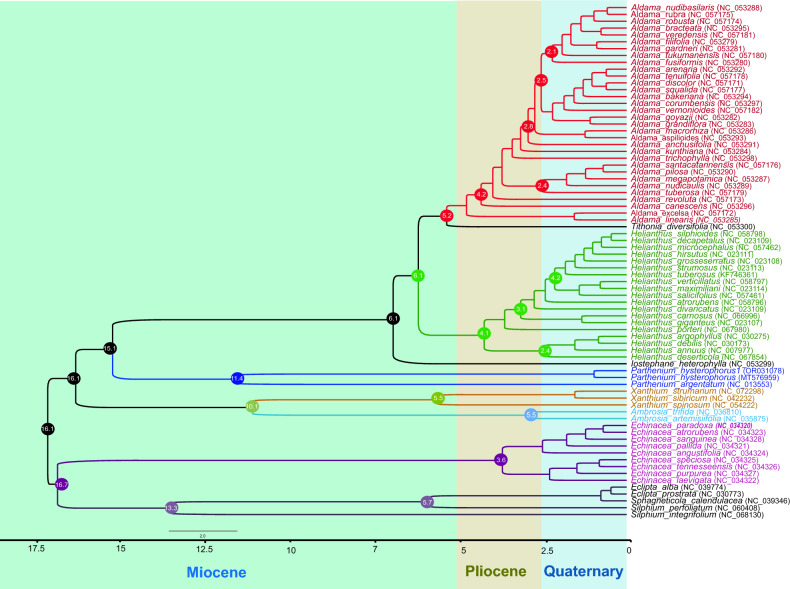
Divergence time estimates of *P. hysterophorus* based on 72 commonly shared genes among 75 members of the Heliantheae tribe, representing 11 different genera. The GTR + G substitution model was used with four rate categories and a Yule tree speciation model was applied with a lognormal relaxed clock model in BEAST. The 95% highest posterior density credibility intervals are shown for the node ages in circles (mya). Numbers indicate date estimates for different nodes. A geological time scale is shown at the bottom of the Fig.

This study conducted a comprehensive analysis to determine the phylogenetic position of *P. hysterophorus* within the Asteraceae family, specifically the Heliantheae tribe, which comprises 75 members of 11 genera. The investigation involved aligning the sequences of 72 shared genes among these members. Two widely used methods, namely maximum likelihood (ML) and Bayesian inference (BI), were employed for phylogenetic analyses to ascertain the evolutionary relationships. Notably, the ML analysis provided valuable insights by assigning bootstrap values to the nodes in the tree. Remarkably, 40 out of the 72 nodes demonstrated a bootstrap value equal to or exceeding 95%, indicating robust support for their placements (Fig. 9). Upon constructing the phylogenetic trees using the 72 shared gene sequences, it was observed that *P. hysterophorus* formed a distinctive clade along with *P. argentatum*. Both bootstrap analysis and Bayesian inference consistently supported this clustering. Analysis of multiple data sets revealed that *P. hysterophorus*, a plant species, shares a close evolutionary relationship with the genera *I. heterphylla* and *Helianthus*. Similarly, *T. deversifolia* was found to be closely related to the *Aldama* genus. Additionally, *Echinacea, Xanthium*, and *Ambrosia* genera were clustered with strong statistical support, indicating their shared evolutionary history. Conversely, the genera *Eclipta, Sphagneticola*, and *Silphium* formed a distinct clade at the base of the phylogenetic tree. Using the Bayesian approach implemented in BEAST, the divergence time between *Parthenium* and *Helianthus* was estimated at approximately 15.1 million years ago (Mya) with a 95% highest posterior density (HPD) interval of 11.2–22.25 Mya (Fig. 10). This analysis also suggested that the Heliantheae tribe, encompassing these plants, diverged around 22–26 million years ago during the early Miocene period. The TimeTree web tool was employed to verify these results further (Fig S3), yielding similar estimates and supporting the findings derived from maximum likelihood (ML) and maximum parsimony (MP) methods.

## Discussion

According to the present study, the complete plastome of *P. hysterophorus* was analyzed, revealing a length of approximately 151.8 kilobase pairs (kbp) (Table 1 and Fig. 1). Like other angiosperms, the *P. hysterophorus* genome displayed a characteristic quadripartite structure (Fig. 1). In terms of gene content, the *P. hysterophorus* chloroplast genome was found to encode around 129 genes , comprising 85 protein-coding genes, eight ribosomal RNA genes, and 36 transfer RNA genes. Additionally, the genome exhibited 40 microsatellites scattered randomly throughout its sequence. Furthermore, the study identified various types of repeats in the *P. hysterophorus* chloroplast genome. Approximately 14 forward, 45 tandem, 18 reverse, and 16 palindromic repeats were detected (Fig. 7). The findings about the gene content and repetitive elements in the chloroplast genome of *P. hysterophorus* align with previously reported observations in other members of the Asteraceae family, including *P. argentatum*^23^, *Helianthus annuus*^21^, *Helianthus giganteus*^33^, as well as other related species^34^. The protein-coding gene known as *rps*12 exhibits an uneven distribution within the genome. Specifically, its 5' terminal exon is situated in the large single-copy (LSC) region, while two copies of the 3' terminal exon and intron are found within the inverted repeats (IRs). This distribution pattern of *rps1*2 is consistent with observations made in other angiosperm plastomes^34,35^. Hence, the positioning of *rps*12 exons and introns in different regions of the chloroplast genome is a phenomenon shared among various flowering plant species.

In the chloroplast genome of *P. hysterophorus*, we found fifteen genes with introns. Thirteen genes had a single intron, while *ycf*3, *clp*P, and *rps*12 had two introns each. The longest intron was observed in the *rpo*C1 gene, spanning 1,636 base pairs, followed by the *ndh*B gene with an intron length of 776 base pairs. These introns are crucial for regulating gene expression. Recent studies indicate that strategically positioned introns can boost the expression of introduced genes^36^. Thus, introns can serve as valuable tools for improving the efficiency of genetic transformation. Interestingly, it has been noted that genes such as *ycf*1, *ycf*2^37,38^, *rpl*23^39^, and *acc*D^40,41^ are often absent in plant genomes. However, these genes were detected in the reported *P. hysterophorus* plastomes, consistent with findings in other members of the Asteraceae family^41,42^.

We have identified 93 repeat sequences in the chloroplast (cp) genomes of *P. hysterophorus*. These repeats consist of reversed, forward, tandem, and palindromic sequences. Repeat sequences are highly valuable in studying the evolutionary relationships of species^43,44^. They also play a significant role in genome rearrangements^44^. Previous investigations of various plastomes have demonstrated the essential role of repeat sequences in causing insertions and substitutions^45,46^. In the case of *P. hysterophorus*, the length of the identified repeats was relatively short, ranging from 11 to 20 base pairs. Similar results have been reported in plastomes of other plant species from the Asteraceae family^21,23,34^. However, longer repeats have been observed in other plant families, such as a 132-base pair repeat in Poaceae and a 287-base pair repeat in Fabaceae^47^. The presence of longer repeats in DNA sequences can significantly contribute to sequence variation and rearrangement within the genome. This phenomenon occurs through mechanisms like slipped strand mispairing and improper recombination, as extensively discussed earlier^21,48^. These repeats, which are characterized by the repetition of specific DNA segments, have been identified as significant hotspots for genome reconfiguration, highlighting their crucial role in shaping genetic landscapes^48^. Moreover, the importance of these repetitive elements extends beyond their impact on genomic stability. They also serve as invaluable resources for developing genetic markers utilized in various studies involving the phylogenetics and population analysis of *P. hysterophorus* and its closely related species.

We extensively analyzed perfect simple sequence repeats (SSRs) within the plastome of *P. hysterophorus,* and a comparative analysis was undertaken with ten closely related species belonging to the Helianthae tribe.. SSRs are specific regions of DNA that tend to undergo mutations at a higher rate due to the slipping of DNA strands. These regions exhibit significant variation in the number of repeat units within the chloroplast genome, making them valuable molecular markers for studying plant population genetics, evolution, and ecology^49^. In our study, we focused on identifying SSRs that were ten base pairs or longer, as these have been suggested to be more susceptible to slipped strand mispairing, which is considered the primary mechanism for generating SSR polymorphisms^50,51^. Our investigation revealed the presence of 40 SSRs in the plastome of *P. hysterophorus*, exclusively comprising 100% mononucleotide SSRs. Furthermore, SSRs with repeat motifs 37, 38, 45, and 46 were identified in the plastomes of *P. argentatum*, *E. angustifolia*, *S. integrifolium*, and *X. sibiricum*, respectively. These findings align with previous research indicating that chloroplast genome SSRs are predominantly composed of mononucleotide repeats of 'A' or 'T'^52,53^. Our research findings are in line with previous studies that have consistently highlighted the prevalence of polythymine (polyT) or polyadenine (polyA) repeats in plastomes. These repetitive patterns of short sequence repeat (SSRs) have been observed to be more abundant compared to tandem cytosine (C) and guanine (G) repeats, which are relatively less common^54,55^. The presence of polyT or polyA repeats contributes significantly to the overall composition of plastomes in *P. hysterophorus*. This observation is consistent with earlier investigations across different species, indicating a high proportion of 'AT' base pairs^23,56^. Such 'AT'-rich regions have also been reported in previous studies, emphasizing the correlation between repetitive patterns and the prevalence of 'AT' base pairs in plastomes.

According to genome synteny and comparison analysis, the plastome of *P. hysterophorus* shows significant sequence similarity with other species belonging to the Heliantheae tribe (Fig). This analysis also confirms the presence of a rearrangement in the large single-copy region (LSC), involving a double inversion spanning 25 kb, which has been previously reported in other members of the Asteraceae and a few other families^23,57–59^. We identified substantial sequence congruence between *P. hysterophorus* and its closely related species. Nevertheless, our comprehensive sequence analysis also unveiled noteworthy divergences within specific genomic regions. These variations resulted in relatively lower identity between the species in these comparable regions. Furthermore, consistent with previous findings on plastomes of related species^35,46,60,61^, the LSC and SSC regions exhibited lower similarity compared to the two inverted repeat (IR) regions in all the studied species' plastomes. This suggests that the IR regions are more conserved across these species.

Previous research has yielded consistent outcomes when examining various higher plant species' plastomes (plastomes). These outcomes indicate that there is a distinct pattern of sequence divergence within the plastomes, particularly in the inverted repeat (IR) regions, as compared to the small single-copy (SC) and large single-copy (LSC) regions. This discrepancy in sequence divergence is likely due to a fascinating phenomenon called gene conversion, which involves the correction of genetic copies between IR sequences. In other words, the IR regions display a remarkably lower degree of sequence variation, suggesting that gene conversion is a mechanism for maintaining genetic integrity and homogeneity within these regions^62^. Furthermore, an interesting observation was made regarding the non-coding regions, which displayed a significantly higher level of divergence than the coding regions. This finding indicates that these non-coding regions have undergone substantial variations over time, suggesting a potential role in shaping genetic diversity. Specifically, the following regions and genes displayed significant divergence*: trn*H-*psb*A, *mat*K, *rps*16-*trn*E, *trn*R-*psb*D, *ndh*C-*trn*V, *ycf*3-*trn*S, *clp*P, *pet*B, *ycf*1, *rpo*A, *rpl*32, and *ndh*F^60,61^. These findings align with previous studies^35^ and confirm the existence of similar differences among various coding regions in the species analyzed. Furthermore, the results support the notion that these divergent genes are predominantly located in the LSC regions and exhibit a tendency toward faster evolution^34^.

The expansion and contraction at the borders of inverted repeats (IRs) are major factors contributing to size variations among plastomes, playing a crucial role in evolution^63–65^. In order to investigate these variations, a comprehensive analysis was conducted on the two IRs and two single-copy regions of the plastomes of *P. argentatum*, *A. anchusifolia*, *A. artemisiifolia*, *E. angustifolia*, *H. annuus*, *I. heterophylla*, *T. diversifolia*, *S. integrifolium*, *S. calendulacea*, and *X. sibiricum*, in comparison to*P. hysterophorus.* Notably, no significant differences were observed in the length of the IRs among these plastomes. However, certain genes at the junctions of the IRs and single-copy regions, such as *rps*19, *ycf*1, and *rpl*2, exhibited slight variations (Fig. 6).

Previous studies have extensively used plastid genes to support the monophyly of Asteraceae^66^. These studies have also identified 45 tribes within the family, organized into 13 subfamilies^1,67^. Plastid sequences have been crucial in determining the relationships between Asteraceae subfamilies and most tribes^68,69^. However, some uncertainties still exist in these relationships. The utilization of plastome genomes in phylogenetic studies and molecular evolutionary systematics has yielded immense value by offering a profound comprehension of intricate evolutionary connections within the realm of angiosperms. This avenue of research has provided researchers with a comprehensive understanding of the complex relationships that exist among various species of flowering plants^34,68–71^. Consequently, in this study, we utilized 72 shared protein-coding genes from 75 representatives of 11 genera to establish the phylogenetic position of *P. hysterophorus* within the tribe Heliantheae. Both Bayesian inference (BI) and maximum likelihood (ML) methods were employed for the phylogenetic analysis (Fig. 9). The study's results revealed that *P. hysterophorus* and *P. argentatum* are closely related, which was strongly supported by reliable statistical measures like a 100% bootstrap value and Bayesian inference. This close relationship was determined through the analysis of phylogenetic studies carried out by^72^. Additionally, the position of *P. hysterophorus* within Heliantheae, as confirmed by this study, aligns with the previously published phylogeny described^72,73^. According to a Bayesian approach implemented in BEAST, the estimated divergence time between *Parthenium* and *Helianthus* is approximately 15.1 million years ago (Fig. 10). Furthermore, the tree generated by BEAST exhibited a consistent topology with those produced by maximum likelihood (ML) analysis. These findings were also corroborated by a study conducted by^72^ on the basis of transcriptomics data. Our findings align with the results obtained from TimeTree, which indicated that the adjusted time divergence between *Parthenium* and *Helianthus* occurred approximately 15.0 million years ago (Mya) (Fig. 10 and Fig S3). These results are in line with previous reports on the estimation of the divergence time of the Helianthae tribe (Fig S3).

## Materials and methods

### Chloroplast DNA extraction, sequencing, and assembly

To extract high quality DNA from young and immature leaves of *P. hysterophorus*, we employed a meticulous process. Firstly, the leaves were finely ground into a fine powder using liquid nitrogen. This method ensured that the DNA would be released from the cells effectively. To isolate the DNA, we utilized the highly reliable DNeasy Plant Mini Kit from Qiagen (Valencia, CA, USA). This kit provided us with a robust and efficient method for DNA extraction from plant samples. The kit's protocol was followed carefully to obtain high-quality DNA. Once the DNA was successfully isolated, we proceeded to sequence the chloroplast DNA using an Illumina HiSeq-2000 platform at Macrogen (Seoul, Korea). This cutting-edge sequencing platform allowed us to generate a vast amount of raw reads for *P. hysterophorus*, specifically around 475,610,881 raw reads. However, to ensure the reliability and accuracy of our analysis, we needed to filter out low-quality sequences. To achieve this, we implemented a stringent filtering criterion based on a Phred score of less than 30. This quality control step eliminated any reads that did not meet the desired threshold, ensuring that only high-quality sequences were retained for further analysis. To assemble the plastomes with precision, we employed two different methods. Firstly, we utilized the GetOrganelle v 1.7.5 pipeline^74^, which is a sophisticated tool specifically designed for plastome assembly. Additionally, we also employed SPAdes version 3.10.1 (http://bioinf.spbau.ru/spades) as an assembler to enhance the accuracy and reliability of the assembly process.

### Genome annotation

The annotation process of the plastomes involved several steps using established tools and software. CpGAVAS2^75^ and DOGMA (http://dogma.ccbb.utexas.edu/, China)^76^, widely recognized online tools for genome annotation, were utilized to carry out the initial annotation. Additionally, tRNAscan-SE^77^, a well-established program, was employed to identify tRNA genes within the plastomes. To ensure the accuracy of the annotations, a comparative analysis was conducted by comparing the plastomes with reference genomes using Geneious Pro v.10.2.3^78^ and tRNAs can-SE (v.1.21)^77^. This step allowed for the identification of start and stop codons, determination of intron boundaries, and implementation of manual alterations when necessary. To visualize the structural features of the plastomes, chloroplot, a powerful tool developed by^79^, was used. Furthermore, the genomic divergence was assessed using mVISTA in shuffle-LAGAN mode, with the plastome of *P. hysterophorus* serving as the reference^80^. In the *P. hysterophorus* plastome, the average pairwise sequence divergence with eleven related species (*P. hysterophorus* (old), *P. argentatum*, *A. anchusifolia*, *A. artemisiifolia*, *E. angustifolia*, *H. annuus*, *I. heterophylla*, *T. diversifolia*, *S. integrifolium*, *S. calendulacea* and *X. sibiricum*) from the tribe Heliantheae was determined. We extensively compared gene order and performed multiple sequence alignment. This allowed us to employ comparative sequence analysis to identify any missing or unclear gene annotations. For whole genome alignment, we used MAFFT version 7.222 with default parameters^81^. Pairwise sequence divergence was calculated using Kimura’s two-parameter (K2P) model. This approach ensured accurate assessment of the genetic data. In our analysis, we employed the DnaSP software version 6.13.03^82^ to perform a sliding window analysis with a window size of 200 bp and a step size of 100 bp. This analysis allowed us to calculate nucleotide variations, specifically the nucleotide diversity (Pi). In order to visualize the shared genes and genes divergence among different species plastomes, we utilized the heatmap2 package in the R software. Additionally, we created a synteny plot using the pyGenomeViz version 0.2.1 package, employing the pgv-mmseqs mode and setting an identity threshold of 50%. The relevant source for pyGenomeViz can be found on GitHub at the following URL: https://github.com/moshi4/pyGenomeViz.

### Characterization of repetitive sequences and SSR

The analysis of tandem repeats was conducted using Tandem Repeats Finder version 4.07, following the default settings described by^83^. For microsatellite analysis of *P. hysterophorus* and eleven other related species plastomes, the MIcroSAtellite (MISA) identification tool was utilized^84^. The minimum distance between two SSRs (Simple Sequence Repeats) was set to 100 base pairs. To identify the SSRs, we employed the following search parameters: pentanucleotide and hexanucleotide repeats required a minimum of three repeat units, trinucleotide and tetranucleotide repeats required a minimum of four repeat units, dinucleotide repeats required a minimum of eight repeat units, and mononucleotide repeats required a minimum of ten repeat units. REPuter software^85^ was used to identify repetitive sequences (such as palindromic, reverse, and direct repeats) within the twelve plastomes, namely *P. hysterophorus*, *P. hysterophorus* (old), *P. argentatum*, *A. anchusifolia*, *A. artemisiifolia*, *E. angustifolia*, *H. annuus*, *I. heterophylla*, *T. diversifolia*, *S. integrifolium*, *S. calendulacea*, and *X. sibiricum*. The repeat identification settings in REPuter were as follows: a minimum repeat size of 30 base pairs, ≥ 90% sequence identity, and a Hamming distance of 1.

### Sequence divergence and phylogenetic analysis

In order to explore the evolutionary connection of *P. hysterophorus* within the Heliantheae tribe, a comprehensive analysis was conducted using a dataset comprising 72 commonly shared genes among 75 members of the Heliantheae tribe, representing 11 different genera. To ensure accuracy, the nucleotide sequences of these 72 protein-coding genes were aligned and combined using MAFFT, employing the default settings as outlined by^86^. The best-fitting model of nucleotide evolution, TVM + F + I + G4, was determined by jModelTest 2^87^. Two distinct approaches were employed to deduce the phylogenetic relationship of *P. hysterophorus*. Firstly, a Bayesian inference (BI) tree was constructed using Mrbayes 3.12, utilizing the Markov chain Monte Carlo sampling method. Secondly, a maximum likelihood (ML) tree was generated using PAUP* 4.0^88^. The ML tree was created by running 1000 bootstraps, which provided support values for different nodes. For the BI analysis, a total of four chains were employed: three heated chains and one cold chain. These chains were run for 10,000,000 generations, with a sampling frequency of 1000 and a print frequency of 10,000. To ensure convergence, a burn-in of 2500 (25% of the total number of generations divided by the sampling frequency) was implemented. Finally, a 50% majority-rule consensus tree was derived from the phylogenetic trees generated, and Figtree^89^ was utilized to visually represent the relationships among the moss species based on their plastome sequences.

To determine when *P. hysterophorus* diverged from 75 other members, we used a concatenated data matrix in BEAST^90^. In our analysis, we utilized a substitution model known as general time reversible (GTR + G), which incorporates four rate categories. Additionally, we employed a Yule tree speciation model and a lognormal relaxed clock model. To determine the molecular divergence, we employed an average substitution rate of 3.0 × 10^−9^ substitutions per site per year (s/s/y) derived from a fossil-based approach. Unfortunately, the fossil record in the Helianthae group is limited, and the few fossils available cannot be confidently assigned to any existing genera. As a result, we employed an alternative calibration approach. To assess the effectiveness of our approach, we examined the data by combining protein-coding genes. We utilized an online tool called TimeTree (http://www.timetree.org/)^91^, to estimate divergence times and make the final determination (Fig. S3). In our dating studies, we conducted three separate Markov chain Monte Carlo (MCMC) runs, each consisting of 50 million generations. To ensure reliability, we combined the tree files from all three runs using LOGCOMBINER. Convergence and adequate sample sizes were assessed using TRACER 1.5^92^. We discarded the first 25% of trees in each analysis to eliminate potential bias. Finally, we constructed the tree using TREEANNOTATOR and utilized FIGTREE 1.4 to visualize the tree, with a 95% highest posterior density (HPD) interval.

### Ethics approval and consent to participate

The authors declared that experimental research works on the plant described in this paper comply with institutional, national and international guidelines. Field studies were conducted in accordance with local legislation and get permissions from provincial department of forest of and grass of Khyber pakhtunkhwa province, Pakistan.

## Conclusion

In this studywe sequenced and analyzed the complete chloroplast genome of *P. hysterophorus* and compared it to related species in the Asteraceae family. Our analysis revealed that the chloroplast genome of *P. hysterophorus* encompasses a total length of 151,881 bp. Structural similarities and intriguing variations were found when comparing the *P. hysterophorus* plastome to those of related species. Moreover, a number of different genes, including *mat*K, *ndh*F, *clp*P, *rps*16, *ndh*A, *rps*3, and *ndh*D, showed significant gene divergence in our analysis. The analysis has provided evidence supporting the presence of a rearrangement (inversions) in the LSC region of the plastome. The phylogenetic analysis revealed that *P. hysterophorus* shares a close evolutionary relationship with the genera *I. heterphylla* and *Helianthus*. The divergence time between Parthenium and Helianthus was estimated at approximately 15.1 million years ago (Mya). Our findings provide valuable insights into the genetic characteristics and evolutionary history of *P. hysterophorus*. This study contributes to our understanding of the plastomes in the Asteraceae family and can serve as a valuable resource for further research on *P. hysterophorus* and related species.

### Supplementary Information

Supplementary Figures.

Supplementary Table S1.

## Data Availability

All data generated or analyzed during this study are included in this published article. *P. hysterophorus* plastome was submitted to NCBI with accession number (OR031078).
