# Publisher Correction: The complete plastome sequences of invasive weed *Parthenium hysterophorus*: genome organization, evolutionary significance, structural features, and comparative analysis

**DOI:** 10.1038/s41598-024-55391-0

**Published:** 2024-02-27

**Authors:** Lubna, Sajjad Asaf, Rahmatullah Jan, Saleem Asif, Saqib Bilal, Abdul Latif Khan, Ahmed N. Al-Rawahi, Kyung-Min Kim, Ahmed AL-Harrasi

**Affiliations:** 1 Natural and Medical Sciences Research Center, University of Nizwa, 616 Nizwa, Oman; 2 Department of Applied Biosciences, Kyungpook National University, Daegu, 41566 Republic of Korea; 3 Department of Engineering Technology, University of Houston, Sugar Land, TX 77479 USA

Correction to: *Scientific Reports* 10.1038/s41598-024-54503-0, published online 18 February 2024

The original version of this Article contained an error in Figure 10, where the names in the lower part of the Phylogenetic tree did not display correctly.

The original Figure 10 and accompanying legend appear below.

**Figure 10:**
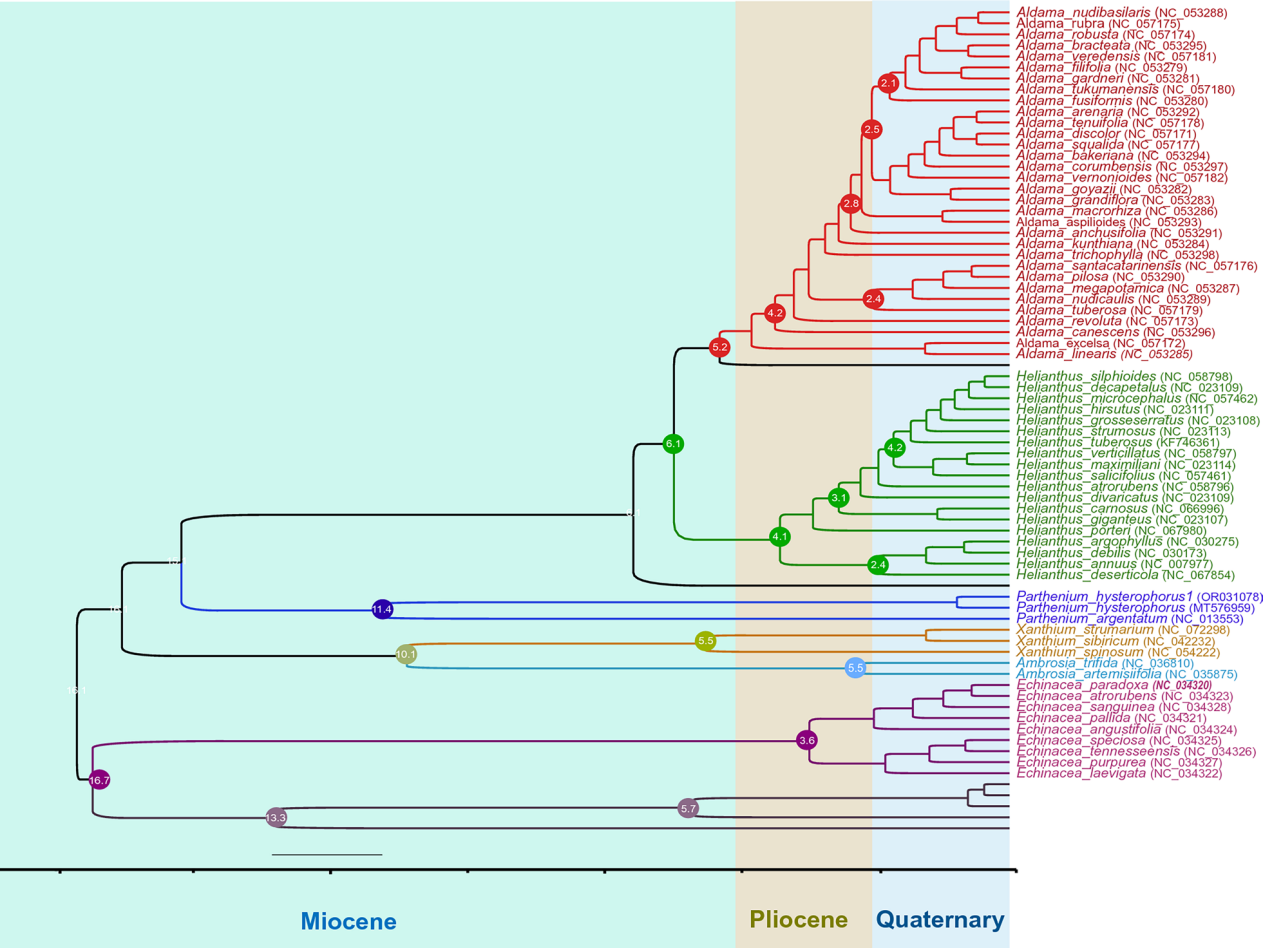
Divergence time estimates of *P. hysterophorus* based on 72 commonly shared genes among 75 members of the Heliantheae tribe, representing 11 different genera. The GTR + G substitution model was used with four rate categories and a Yule tree speciation model was applied with a lognormal relaxed clock model in BEAST. The 95% highest posterior density credibility intervals are shown for the node ages in circles (mya). Numbers indicate date estimates for different nodes. A geological time scale is shown at the bottom of the Fig.

The original Article has been corrected.

