# Supplementary material for: The complete plastome sequences of invasive weed Parthenium hysterophorus: genome organization, evolutionary significance, structural features, and comparative analysis
Source: Sci Rep. 2024 Feb 18;14:4006. doi: 10.1038/s41598-024-54503-0 (PMC10874969; doi:10.1038/s41598-024-54503-0)
Supplement: Supplementary file 1 — Supplementary Figures. [file 41598_2024_54503_MOESM1_ESM.docx]

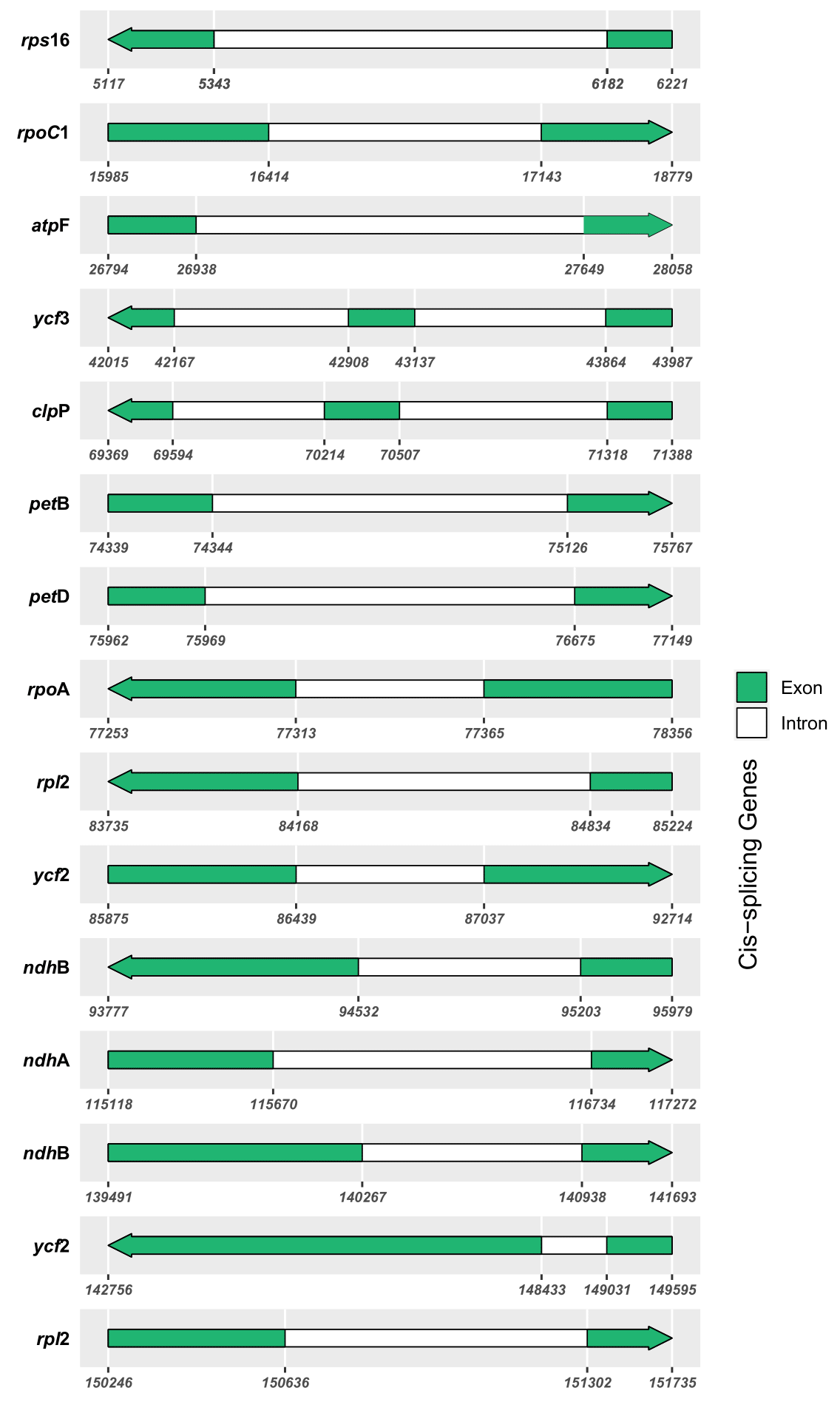


**Fig S1.** Introns and exons lengths for the splitting genes in plastomes of *P. hysterophorus.*


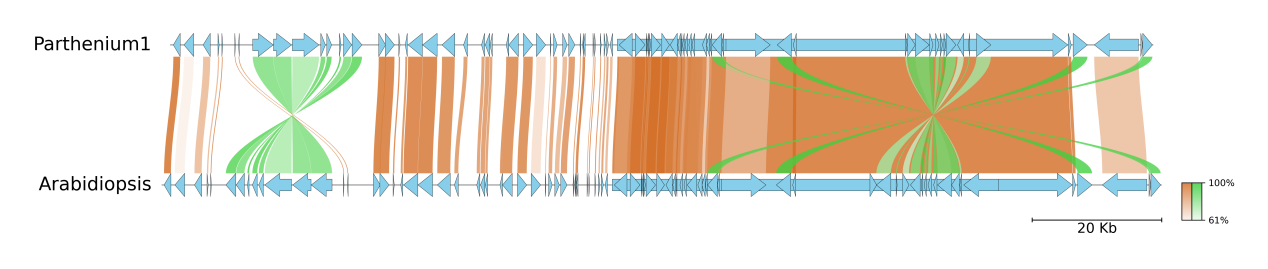


**Fig S2.** Synteny plot of *P. hysterophorus* and P. argentatum plastomes. The synteny plot shows normal links with chocolate color, inverted link with lime-green color, and gene feature with sky-blue color.


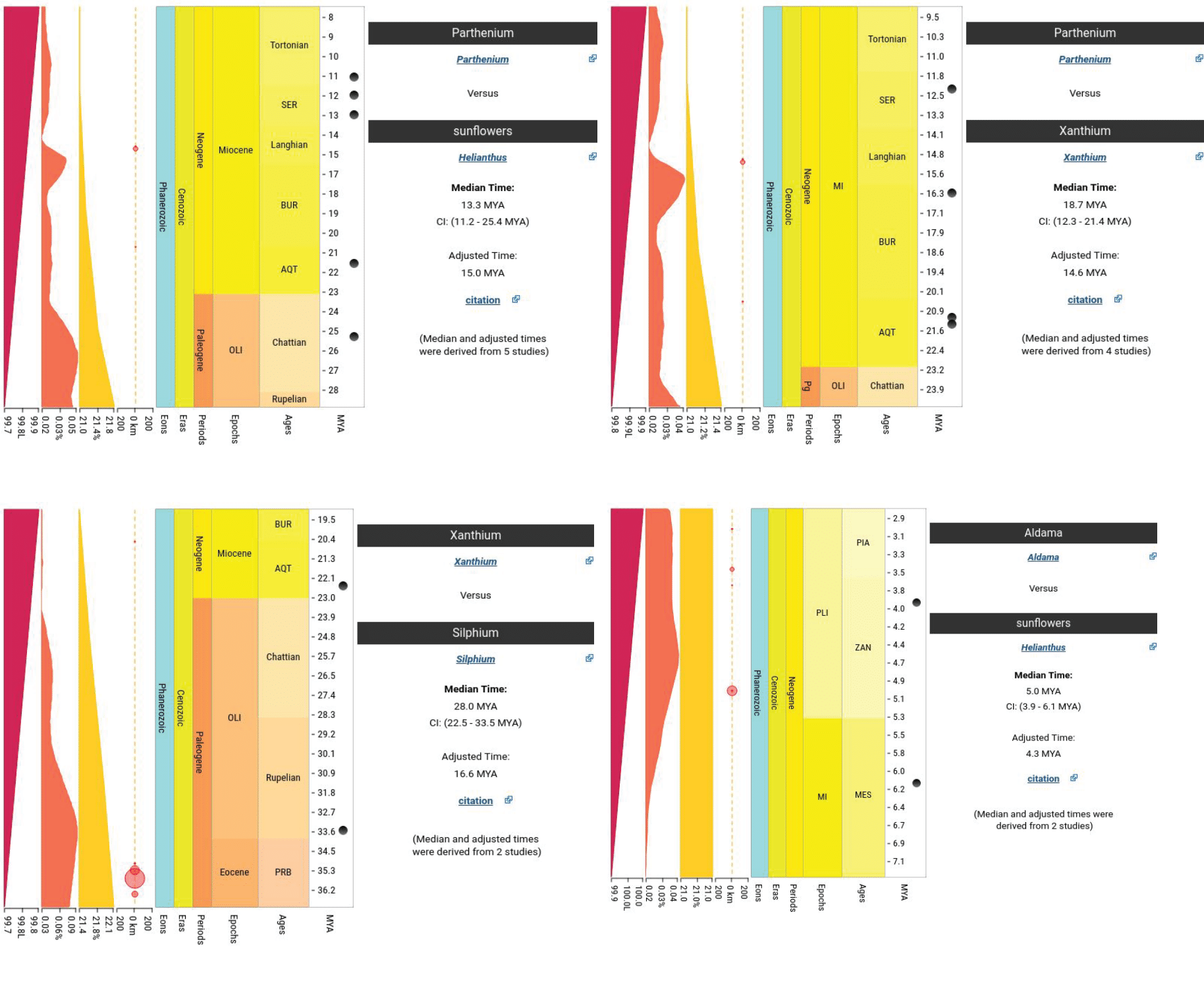


**Fig S3.** The divergence time estimation between *Parthenium* vs *Helianthus*, *Parthenium* vs *Xanthium*, *Xanthium* vs *Silphium* and *Aldama* vs *Helianthus* based on TimeTree.
